# Non-resolving Pneumonia As Presentation of Incomplete Kawasaki Disease in Arabic Girl

**DOI:** 10.7759/cureus.17280

**Published:** 2021-08-18

**Authors:** Ali S Alsarhan, Walid M Abuhammour

**Affiliations:** 1 Pediatric Infectious Diseases, Al Jalila Children's Specialty Hospital, Dubai, ARE

**Keywords:** kawasaki disease (kd), incomplete kawasaki disease, non-resolving pneumonia, intravenous immunoglobulins (ivig), systemic steroids

## Abstract

Kawasaki disease (KD), particularly incomplete form, might present with wide spectrum clinical features. The treatment regimen includes a combination of intravenous immunoglobulins (IVIG) and aspirin. The use of steroids has been studied as an adjunctive therapy and its role in preventing coronary artery (CA) complications is still debatable. Here, we are presenting a rare presentation of incomplete KD.

A previously healthy 5-year-old Arab girl, presented with clinical features consistent with pneumonia, rash, and enlarged cervical lymph nodes. On admission, antibiotics were administered intravenously in addition to steroids considering her reactive airway disease history which resulted in interim improvement. Yet, upon clinical worsening, her clinical status was revised, laboratory and physical examination revealed raised inflammatory markers, new opacity of pulmonary consolidation on chest X-ray, and peeling of skin. Because of high clinical suspicion of incomplete KD combined with her echocardiography that showed prominent coronary arteries, diagnosis of incomplete KD was made. After treating her with IVIG and aspirin, the patient made a full recovery.

We are reporting pneumonia-like presentation of incomplete KD. High index of suspicion is required to diagnosis and treat promptly to prevent complications.

## Introduction

Kawasaki disease (KD) is a vasculitis pathology that can damage the coronary arteries (CA) permanently [1], hence, a prompt diagnosis and intervention are needed. Given the fact that criteria for diagnosing KD were not met on many occasions, cases were labeled as incomplete KD, resulting in delayed management. Few of these cases showed atypical presentations involving unusual organs; resulting in more challenges to diagnose and intervene early.

KD criteria include several manifestations, at which the patient must present with at least four of them. These manifestations include bilateral non-purulent conjunctivitis, cervical lymphadenopathy, polymorphic rash, extremities and oral mucosal alterations. Fever for at least 5 days is mandatory to establish the diagnosis. High index of suspicion, despite failure to meet the criteria, can promote the diagnosis of incomplete KD by an expert [1].

According to the American Heart Association, the mainstay of treatment for KD and incomplete KD is intravenous immunoglobulins (IVIG), which must be given at the earliest possible. In addition to IVIG, moderate to high dose of acetylsalicylic acid (aspirin) is administered to suppress the inflammation despite lack of evidence supporting the role of aspirin in mitigating CA complications. Other treatment modalities are recommended in the management of IVIG-resistant KD cases; which include steroids, biological agents, and plasmapheresis. Controversy exists debating the benefits of steroids in preventing CA complications [2].

## Case presentation

A 5-year-old girl presented to Emergency Department (ED) in June 2019 mainly with dry cough and fever for 5 days duration. Her complaints were associated with generalized rash involving the body and the cheeks, fatigue, vomiting, and loose bowel motion prior to her presentation. Other aspects of the patient’s history were unremarkable apart from penicillin allergy and reactive airway disease (RAD). Upon initial assessment, she was found to be normotensive, normothermic, but tachypneic. Basic laboratory workup (Table 1) showed leukocytosis, microcytic hypochromic anemia, and elevated inflammatory markers (C-reactive protein (CRP) and procalcitonin).

**Table 1:** Timeline changes of blood test results. WBC: white blood cells; MCV: mean corpuscular volume; MCH: mean corpuscular hemoglobin; CRP: c-reactive protein; ESR: erythrocyte sedimentation rate

Lab test/time	Day 1	Day 2	Day 7	Day 9	Day 12	Day 16
WBC (6,000-15,000 cells/mcl)	17,930	-	32,560	45,060	25,020	10.86
Neutrophils (25-55 %)	86.90	-	-	78	67.3	59.4
Lymphocytes (33-65%)	10	-	-	14.8	23.02	34.8
Hemoglobin (11-14 g/dl)	10.3	-	10.2	9.5	9	8.6
MCV (75-87 fl/cell)	68	-	67.7	68	69.5	70.4
MCH (24-30 pg/cell)	23.5	-	23.4	23.3	23.4	23.4
Platelets (200,000-490,000 cells/mcl)	214,000	-	889,000	1,149,000	1,233,000	780
CRP (<2.8 mg/L)	-	314.31	11.23	209.6	114.38	46.98
ESR (<10 mm/hr)	-	-	-	-	110	117
Procalcitonin (<0.5 ng/ml)	-	20.25	0.5	-	-	-
Ferritin (4-67 ng/ml)	-	-	-	-	546	-

**Figure 1:**
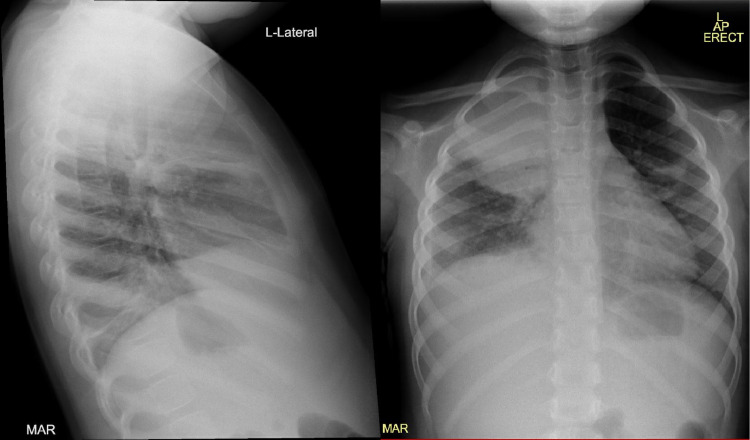
AP and lateral CXR showing right upper lobe consolidation and right pleural effusion. AP: anteroposterior; CXR: chest X-ray

Chest radiogram (CXR) was done, and it revealed right upper zone opacification and right pleural effusion (Figure 1).

Chest ultrasound (U/S) showed moderate size effusion with hypoechoic lesion, central consolidation, and reduced perfusion at the upper right zone. The patient was diagnosed with complex pneumonia and was admitted for IV antibiotics. Oral prednisolone course of 1 mg/kg twice daily was commenced for 5 days due to her history of RAD.

Thorough physical examination revealed an ill-looking child in respiratory distress including mild intercostal retraction and grunting. Chest examination revealed normal air entry. The rest of her examination showed bilateral enlarged non-tender cervical lymph nodes (0.5-1 cm), and erythematous palms and soles. Her cardiovascular, gastroenterological, and neurological systems examination were unremarkable.

During hospitalization, spikes of fever and her respiratory distress persisted necessitating oxygen support by face mask. After infusing IV clindamycin, generalized macular rash erupted again along with erythematous cheeks, soles, and palms. Due to the skin reaction, clindamycin was discontinued, and antibiotics were switched to ceftriaxone (IV) and linezolid (IV). She continued to have mild skin rash, thus, antihistamine was added to her regimen to control it.

Serial studies of chest U/S reported significant improvement after few days with improvement of the perfusion of the right upper zone of the lung. Surprisingly, repeated complete blood count on day 7 showed five folds elevation in the platelets and doubling the leukocytes count despite the sharp drop in the CRP (Table 1).

**Figure 2:**
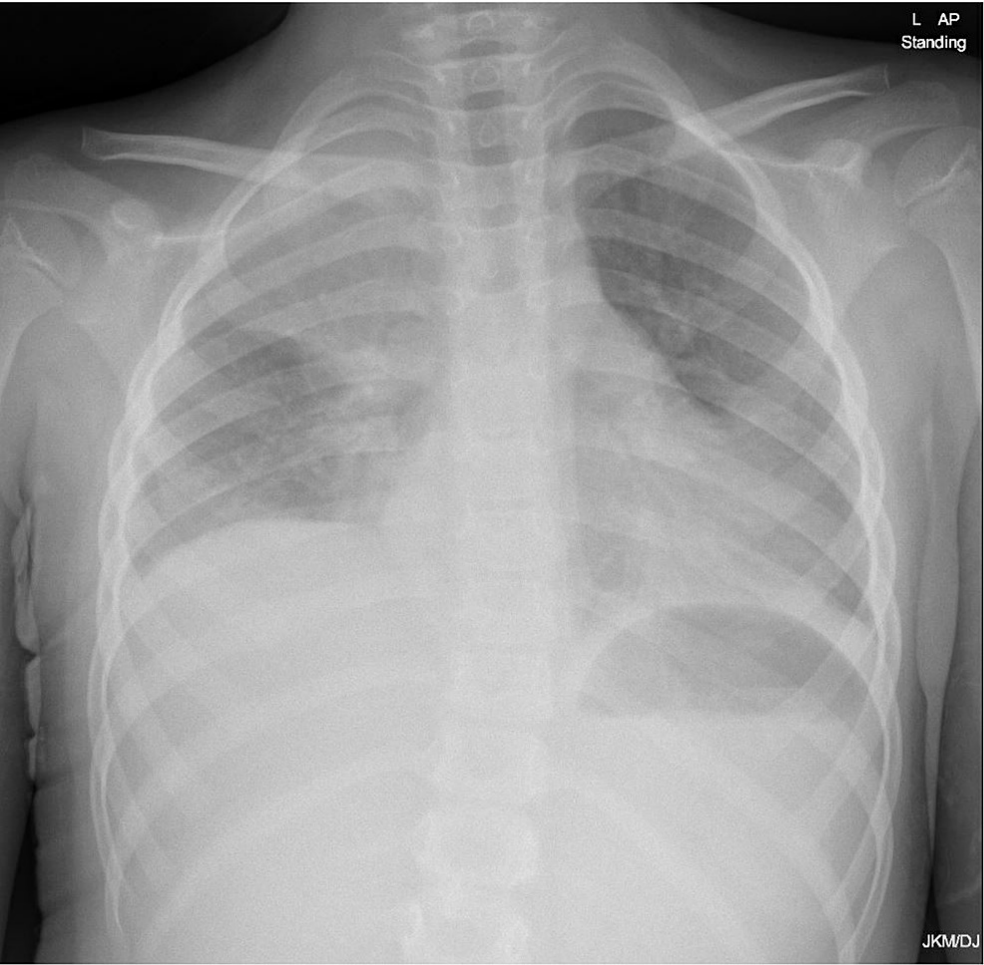
Repeat CXR showing new consolidation on the left side of the lung. CXR: chest X-ray

On day 9, the patient spiked a fever again and started to complain of mild right hypochondrial pain. She was noticed to have fingertips peeling. Abdominal U/S scan was normal, but repeat CXR showed new opacification in the lower zone of the left lung (Figure 2).

Blood tests revealed further elevation in the platelets count, white blood cells count, and CRP level (Table 1). Respiratory viral panel including influenza A/B polymerase chain reaction, and blood culture were all negative to any microorganism. Due to high index of suspicion of KD, echocardiography (ECHO) was performed and interestingly showed prominent CA. IVIG infusion of 2 g/kg was given over 12 hours in addition to high dose aspirin 80 mg/kg divided into four doses. Yet, her temperature didn’t subside after 48 hours mandating a second dose of IVIG.

The patient was discharged after 15 days of hospitalization on low dose aspirin, free of symptoms. The cardiology and the infectious disease teams followed her up at 1 month and 4 months intervals. Her symptoms resolved, repeated blood tests were back to normal (WBC 9.22 cells/mcl, Hb 11.4 g/dl, platelets 450 cells/mcl, ESR 26 mm/hr, CRP 0.72 mg/l), and repeated ECHO reported normal CA.

## Discussion

Incomplete KD is a challenging diagnosis and its signs and symptoms upon presentation might deviate out of the usual (atypical). To the best of our knowledge, only 20 cases of incomplete KD have been reported in the literature who had pneumonia as the initial presentation [3-6]. None of these were reported in Arabic ethnicity. Majority of cases were missed initially and managed improperly due to the lack of high index of suspicion. Reliance on clinical criteria and unavailability of a golden standard test to diagnose incomplete KD resulted in delay of treatment and management. This delay in intervention increased the incidence of CA insult in incomplete KD comparing to complete KD [7].

Lungs can be affected in KD in form of interstitial pneumonitis particularly reticulogranular changes, and pleural effusion [3]. In our case, presence of bronchial breathing, and failure to appreciate crackles at the right upper zone of the lung during the temporary improvement supports our diagnosis of KD rather than pneumonia that evolved into KD. The antibiotics response was proven wrong after re-emergence of the signs and symptoms on day 9, in addition to elevated levels of inflammatory markers and platelet count. We believe that in our case the use of steroids which was given coincidently for the reactive airway disease history helped to mask the disease and mistakenly gave the impression that antibiotics course improved pneumonia. Fingertips peeling, lack of evidence of etiology explaining her condition, and ECHO findings raised the index of suspicion; and thus, made incomplete KD the most likely diagnosis.

By analyzing our reported case and the interventions that were taken, we notice that CA were only prominent and didn’t progress to aneurysmal changes despite delay in diagnosis. Early use of steroids in the management plan might have helped to minimize the damage of CA and reduced the risk of developing complications. Chen et al. and Wardle et al, [8,9] have studied the use of steroids in KD and its impact on the consequences related to CA, particularly in high-risk group. Side effects, time to defervescence. and incidence of coronary arteries aneurysm (CAA) were their main interest. The results showed reduced incidence of CAA, quicker defervescence with no significant side effects after introducing the steroids to the initial regimen. This effect can be potentiated if steroids were administered earlier [8,9]. The findings in our case supported the same impression about the benefit of using steroids in KD. Yet, the data published and the observations are not sufficient to draw a conclusion about implementing the use of a short course of steroids as first-line treatment along with IVIG.

## Conclusions

In conclusion, incomplete KD might have different presentation and could involve any system. Pulmonary consolidation with pneumonia-like picture is a rare clinical presentation for incomplete KD. High index of suspicion is required to early recognize and diagnose those who present with pneumonia but fail to respond to standard treatment in the presence of high inflammatory markers and signs suggesting KD. Performing ECHO and commencing IVIG could be carried out as early as possible to prevent serious consequences.
